# Gait dynamics in the wide spectrum of children with arthrogryposis: a descriptive study

**DOI:** 10.1186/s12891-015-0834-5

**Published:** 2015-12-09

**Authors:** Marie Eriksson, Åsa Bartonek, Eva Pontén, Elena M. Gutierrez-Farewik

**Affiliations:** Department of Women’s and Children’s Health, Karolinska Institutet, Stockholm, Sweden; KTH Mechanics, Royal Institute of Technology, KTH BioMEx Center, Royal Institute of Technology, Stockholm, Sweden

**Keywords:** Motion analysis, AMC, Trunk movements, Joint kinetics, Orthoses

## Abstract

**Background:**

Arthrogryposis Multiplex Congenita (AMC) is a heterogeneous condition characterized by multiple joint contractures at birth. Greater movements in the trunk and pelvis during walking have been observed in children with AMC using orthoses compared to those wearing only shoes. This study investigated gait dynamics in children with AMC and identified compensatory mechanisms that accommodate walking.

**Methods:**

Twenty-six children with AMC who walked with orthoses or shoes and a control group consisting of 37 typically-developing children were evaluated in 3D gait analysis. Children with AMC were divided into subgroups based on which joints needed to be stabilized in the sagittal plane; AMC1 used knee-ankle-foot orthoses (KAFOs) with locked knee joints, AMC2 used KAFOs with open knee joints or ankle-foot orthoses, and AMC3 used shoes.

**Results:**

The Gait Deviation Index was lower in AMC groups than in the control group, with the lowest in AMC1. Excessive trunk movements in frontal and transverse planes were observed in AMC2 and especially in AMC1. Lower hip flexion moment was found in AMC1, while AMC2 and AMC3 showed similar hip flexion moments as the control group. Knee extension moments were similar between the groups. In the frontal plane there were only small differences between the groups in hip abduction moment. A joint work analysis indicated greater contribution from the hip muscles to overall positive work in AMC groups, particularly in AMC1, than in the control group.

**Conclusion:**

All AMC groups showed less hip extension than the control group, but hip flexion moment was significantly lower only in AMC1, which can be attributed to their gait strategy with bilateral locked KAFOs. AMC1, who had weak knee extensors, were helped by their locked KAFOs and therefore showed similar knee extension moment as the other groups. This finding, together with their gait patterns, demonstrates the children’s high reliance on hip muscles and presumably trunk muscles to provide propulsion. Our study shows that with adequate orthotic support, children with AMC and even with severe weakness and contractures can achieve walking.

## Background

The primary attribute of Arthrogryposis Multiplex Congenita (AMC) is multiple joint contractures at birth [1]. The incidence is reported from 1 per 3000-5100 newborns [1, 2]. According to Bevan et al. the most commonly reported affected areas in the lower extremities are foot and ankle joints followed by knee and hip joints [3]. Treatment of contractures in a child with AMC commences soon after birth with stretching in combination with splints or serial casting that hold the joint in an optimal position [3], with goals to increase range of motion, preserve and enhance muscle strength [3], and position the joint well biomechanically. During childhood, contractures and deformities tend to recur, and surgical treatment is often necessary to maintain ambulation or improve lower limb function [4–6].

In children with AMC, functional ambulation ability depends on factors such as severity of lower limb deformities and muscle weakness, primarily in hip and knee extensors [5, 7]. As such, knee-ankle-foot orthoses (KAFOs) with locked knee joints or ankle-foot orthoses (AFOs) are often used to enable or improve walking function [4, 5, 8, 9]. Use of KAFOs with open knee joints has also been described [8, 9]. In children with AMC with plantarflexor weakness, use of a carbon fiber spring ankle joint has been considered to improve some gait parameters [10].

Lower activity level [11], functional exercise capacity [9], and higher oxygen cost and thereby less efficient gait, particularly in those walking with KAFOs with open knee joints or AFOs [9], have been reported. Children with AMC who use orthoses have been shown to walk with greater trunk and pelvis movements than those who do not use orthoses [8]. Reduced internal hip abduction moments have been reported in barefoot children walking with excessive lateral trunk movements, attributed to hip abductors weakness [12].

There are to date very few studies reporting gait dynamics (both kinematics and kinetics) in children with AMC. Objectives were therefore to describe gait dynamics in children with AMC in their highest possible level of functional ambulation.

## Methods

Children with AMC born between 1993–2008 who were treated at the Department of Pediatric Orthopaedic Surgery of Karolinska University Hospital, Stockholm or Uppsala University Hospital, were invited to participate. Inclusion criteria were four limb or lower limb deformities or contractures, independent ambulation with or without orthoses, and age 5–18 years. Of the 35 children eligible for inclusion, seven declined participation, one child who used a prosthesis was excluded, and one child was excluded due to lack of kinetic data; thus 26 children participated in the study with median [range] age 10.3 [5.0–17.8] years, weight 30.4 [16.5–98.0] kg, and height 136.8 [103.0–173.0] cm. This study was approved by the Regional Ethical Review Board in Stockholm, Sweden. Informed consent was obtained, verbally from the children and written from the parents.

A control group consisting of 37 typically-developing (TD) children, age 9.7 [5.1–17.6] years, weight 34.1 [17.6–84.1] kg, and height 136.0 [111.0–185.5] cm, was used as reference group.

### Clinical examination and functional ambulation

All participants underwent a physical examination by the same examiner (ÅB), including assessment of passive range of motion [13] and manual muscle strength testing [14] in the lower extremities. Hip flexion, knee flexion, and plantarflexion contractures were defined as >0° from neutral position. Knee hyperextension was defined as ≥10°. No children had dislocated hips at the time of the study. Functional ambulation was assessed according to a five-level scale [15], with and without orthoses (Table 1). Nineteen of 26 children had some upper extremity involvement. No child used any walking aids.

**Table 1:** Groups, orthoses, functional ambulation, joint contractures, muscle strength, and functional ambulation without orthoses

Subject	Group	Orthoses	Functional	Joint contractures (°)			Muscle strength (grade 0–5)						Functional
			ambulation	Hip	Knee	Plantar	Hip			Knee	Ankle		ambulation
				Flexion	Flexion	Flexion	Flexion	Extension	Abduktion	Extension	Dorsiflexion	Plantarflexion	without
				L/R	L/R	L/R	L/R	L/R	L/R	L/R	L/R	L/R	orthoses
1	AMC1	KAFO-LK	III	10/20	30/30	20/20	4/4	3/1	3/3	2/2	0/0	0/0	V
2		KAFO-LK	III	5/5	20/15	-/5	2/2	3/3	2/2	2/2	1/1	1/1	V
3		KAFO-LK-C	III		20/+15^b^	40/50	3/3	4/4	4/4	3/3	0/0	0/0	V
4		KAFO-LK	III		-/10	20/20	4/4	4/4	4/4	3/3	0/0	0/0	V
5		KAFO-LK-C	III		30/30	25/25	3/3	4/4	4/4	3/3	1/1	1/1	V
6	AMC2	KAFO-O-C	III		15/5	10/-	4/4	4/4	4/4	4/3	0/0	0/0	IV
7		AFO-H	II	-/5	+15/+10	5/-	4/4	4/4	4/4	5/5	3/4	4/4	II
8		AFO-FC	II		+10/+10		4/4	4/4	4/4	4/4	4/4	3/3	III
9		AFO-S	III		15/15		4/4	4/4	3/3	4/4	4/4	2/2	IV
10		KAFO-O-C	II	5/-	10/5	15/-	4/4	4/4	4/4	4/4	1/1	2/2	III
11		AFO-H	II	5/-	+20/+20		4/4	4/4	4/4	4/4	4/4	2/2	III
12		AFO-C^a^	II		-/5	-/10	5/5	5/4	5/4	5/4	4/3	5/3	II
13		AFO-S^a^	II		-/5		4/4	4/4	4/4	5/5	5/3	5/3	II
14		AFO-C	II				5/5	4/4	4/4	5/5	4/4	3/3	II
15		AFO-FC	II		-/+10	20/-	5/5	4/4	4/4	4/4	2/2	2/2	II
16	AMC3	Shoes	I	10/10			5/5	4/4	4/4	4/4	4/4	4/4	I
17		Shoes	I		5/10		4/4	4/4	4/4	4/4	4/3	3/3	I
18		Shoes + FO	I		5/-	10/-	5/5	4/4	4/4	4/4	4/4	4/4	I
19		Shoes	I	10/10	+10/+20		5/5	4/4	5/5	5/5	5/5	5/5	I
20		Shoes	I		+10/+10		5/5	5/5	4/4	5/5	4/4	5/5	I
21		Shoes	I		+15/+15		5/5	4/4	4/4	4/4	4/4	4/4	I
22		Shoes	I		-/5		5/5	5/5	5/5	5/5	4/4	5/5	I
23		Shoes + FO	I		-/5		4/4	4/4	4/4	4/4	3/4	4/4	I
24		Shoes	I	-/10	5/5		5/5	5/5	5/5	4/4	4/4	4/4	I
25		Shoes	I				5/5	5/5	5/5	4/4	4/4	4/4	I
26		Shoes	I	-/10	-/+10	5/-	4/4	5/5	5/5	4/4	4/4	5/5	I

*KAFO* knee-ankle-foot orthosis, *LK* locked knee joint, *C* carbon fiber ankle joint, *O* open knee joint with extension stop, *AFO* ankle-foot orthosis, *H* hinged with restricted range of motion, *FC* flexible carbon fiber, *S* solid, *FO* foot orthosis, *I* community ambulators with no need for a wheelchair, *II* community ambulators who require a wheelchair for long distances outdoors only, *III* household ambulators and wheelchairs users outdoors and long distances indoors, *IV* household ambulators and wheelchair users both outdoors and indoors, *V* non-functional ambulation and wheelchair use for mobility, *L* left, *R* right

+ indicates hyperextension

^a^ unilaterally

^b^ hyperextension is prevented by orthosis

### Orthopedic surgical history

Orthopedic surgical history was retrieved from the medical records. Surgery in more than one joint at the same time was considered as multiple procedures.

### AMC subgroups

The current orthotic programme at Karolinska University Hospital contains subgroups of children with walking ability, based on presence of muscle weakness and need for joint stabilization. The subgroups are children who require: 1) knee and ankle stabilization for knee extensor weakness grade ≤3, 2) ankle stabilization for plantarflexor and dorsiflexor weakness grade ≤3, and 3) only foot stabilization.

Five children were designated subgroup 1 (AMC1) and used KAFOs with locked knee joints (KAFO-LK) which stabilize the knee and ankle in all planes. Ten children were designated subgroup 2 (AMC2); eight children used AFOs which stabilize the ankle in all planes and two used KAFOs with open knee joints (KAFO-O) with unrestricted knee flexion which further stabilize the knee in frontal and transverse planes. Six of the 15 children using orthoses had carbon fiber spring ankle joints, two in AMC1 and four in AMC2. Eleven children were designated subgroup 3 (AMC3) with only shoes, of which 2 children had additional FO (Table 1). To improve biomechanical alignment when required, heel height was adjusted in the orthoses and/or shoes based on each participant’s contracture.

### Gait analysis

All children underwent 3D gait analysis using an eight-camera system (Vicon^©^, Oxford, UK) and full-body 35-marker set (Plug-In-Gait) at a self-selected comfortable pace along a 10-m walkway with two embedded force plates (Kistler^©^, Switzerland). Joint kinetic information was computed using inverse dynamics.

### Data analysis

At least three gait cycles per side were collected for each subject. The Gait Deviation Index (GDI), which summarizes lower body kinematic parameters into a multivariate measure of overall gait deviations, was computed. A GDI of approximately 100 reflects normal kinematics and each 10-point reduction represents one standard deviation from normal [16]. GDI was calculated for left and right sides individually. Normal GDI was computed from the lab’s database of 37 TD children.

Kinematic and kinetic parameters were obtained from each gait cycle and averaged for each side. Internal joint moments were normalized to body weight. Joint work was calculated as the time integral of joint power. Positive and negative joint work in the lower limbs was computed, and contributions from the hip, knee and ankle were computed as percentages of total positive work in the lower extremities (hip + knee + ankle work).

Step length, stride length, walking speed, and cadence were non-dimensionalized [17].

### Statistical analysis

The Wilcoxon signed-rank test was used to evaluate differences in GDI between sides. The Kruskal-Wallis test was used to evaluate differences between AMC1, AMC2, AMC3, and the control group, followed-up by a post hoc Mann–Whitney *U* test. Chi-Square test was used to evaluate differences in numbers of performed orthopedic surgery in lower limb and spine between AMC groups. Statistical analyses were carried out using commercially-available software (SPSS 21.0). Significance level was set at *p* < 0.05.

## Results

There was no difference in GDI between sides (*p* = 0.648) in the entire AMC group. The side with the lowest GDI in each participant was used for further data analysis. For the control group, the side for further analysis was chosen randomly. Results for GDI, kinematics, internal joint moments and joint work are reported as median and inter-quartile range, and the results for each joint’s contribution to total positive work are reported as means.

### Description of gait

**Fig. 1:**
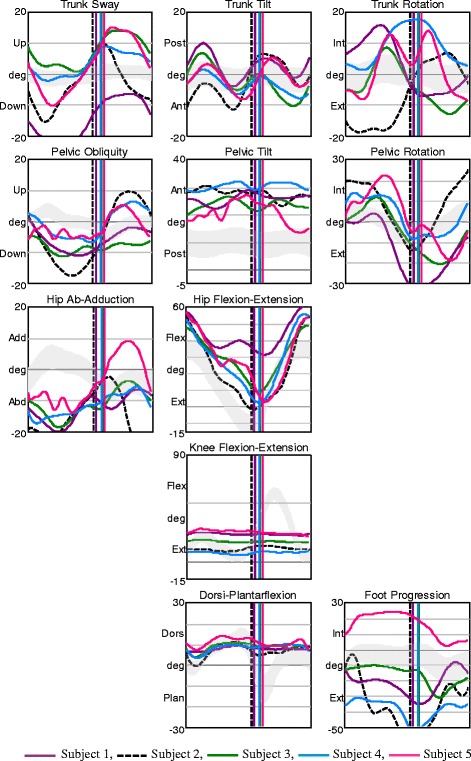
Kinematics in AMC1. Movements in the trunk and pelvis segments, and in the hip, knee, and ankle joints in all planes for AMC1. The shaded field represents the mean ± 1 SD of the gait laboratory control group, and each line represents one individual in this group

**Fig. 2:**
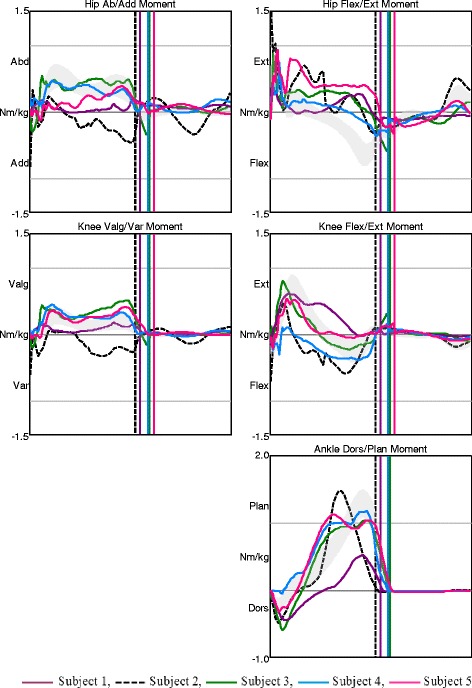
Joint moments in AMC1. Moments in the hip, knee, and ankle joints in the frontal and sagittal planes for AMC1. The shaded field represents the mean ± 1 SD of the gait laboratory control group, and each line represents one individual in this group

All subjects in AMC1 displayed large deviations in trunk and pelvic movements in all planes. They had a constant knee flexion angle as a consequence of the locked orthotic knee joints (Fig. 1). AMC1 displayed prolonged hip extension moment with a late crossover and a short period of low hip flexion moment in late stance. Hip abduction/adduction moment varied among the participants (Fig. 2). Only subject 2 showed a net hip adduction moment instead of abduction moment – this child had hip abductor weakness and walked with approximately 15° trunk lateral sway and nearly 20 ° contralateral pelvic elevation (Figs. 1 and 2, black dashed line). All other had some hip abduction moment during stance (Fig. 2).

**Fig. 3:**
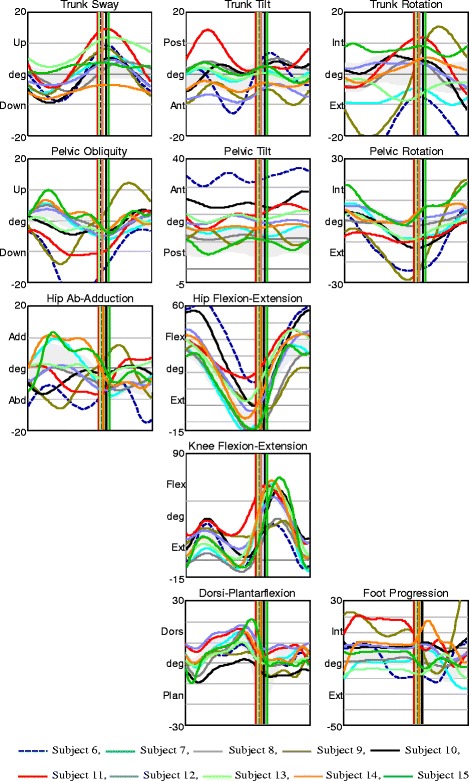
Kinematics in AMC2. Movements in the trunk and pelvis segments, and in the hip, knee, and ankle joints in all planes for AMC2. The shaded field represents the mean ± 1 SD of the gait laboratory control group, and each line represents one individual in this group

**Fig. 4:**
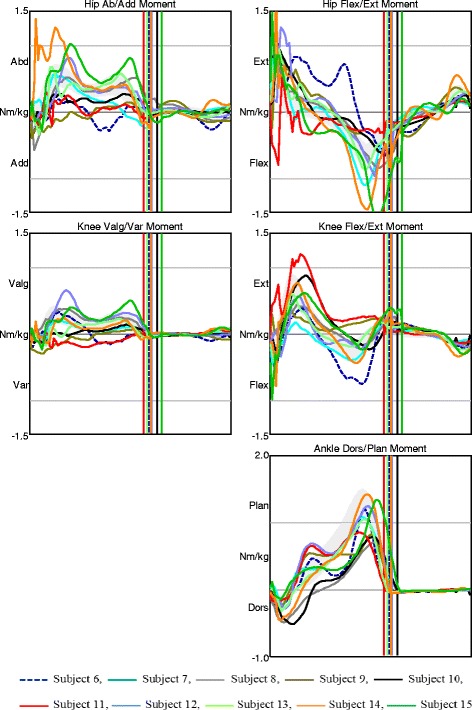
Joint moments in AMC2. Moments in the hip, knee, and ankle joints in the frontal and sagittal planes for AMC2. The shaded field represents the mean ± 1 SD of the gait laboratory control group, and each line represents one individual in this group

There was no characteristic gait pattern in AMC2; we observed a particularly large variation among the participants in hip frontal kinematics, pelvis sagittal kinematics, and in trunk and pelvic rotation (Fig. 3). Hip joint moments and knee joints moments in frontal and sagittal planes also varied within the group (Fig. 4). One participant, subject 6, showed large deviations in lateral trunk sway and in trunk and pelvic rotation. This child had a late crossover from hip extension to hip flexion moment – with an anterior pelvic tilt of approximately 35 °, he did not take advantage of his available hip extension, and had a large range of trunk tilt movement (Figs. 3 and 4, dark blue dashed line).

**Fig. 5:**
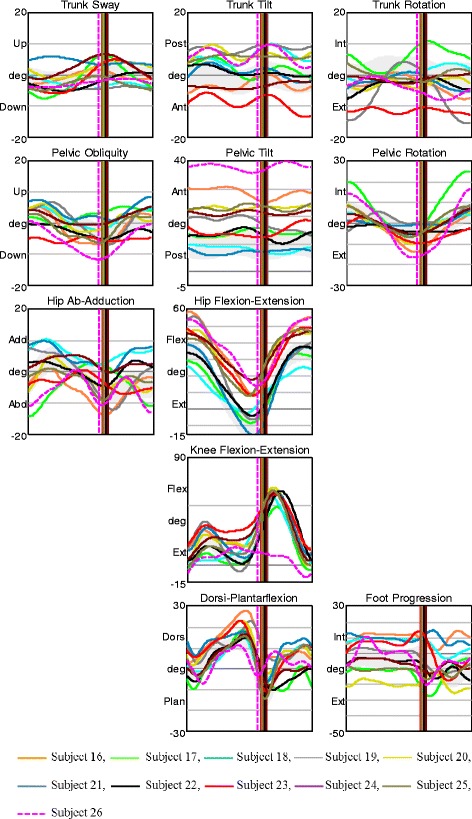
Kinematics in AMC3. Movements in the trunk and pelvis segments, and in the hip, knee, and ankle joints in all planes for AMC3. The shaded field represents the mean ± 1 SD of the gait laboratory control group, and each line represents one individual in this group

**Fig. 6:**
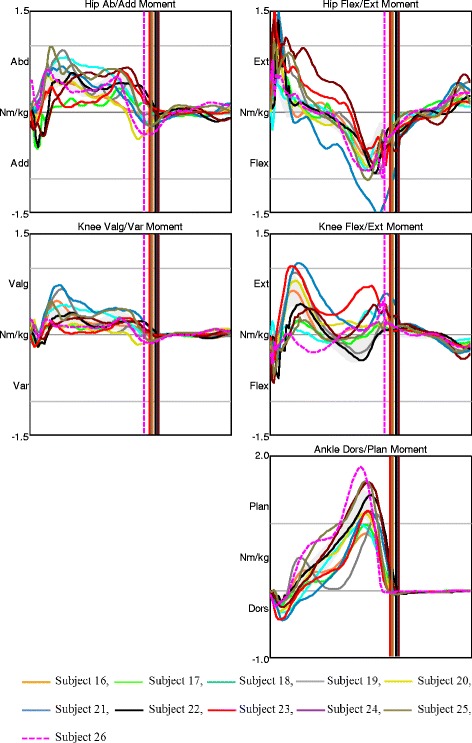
Joint moments in AMC3. Moments in the hip, knee, and ankle joints in the frontal and sagittal planes for AMC3. The shaded field represents the mean ± 1 SD of the gait laboratory control group, and each line represents one individual in this group

The AMC3 group displayed the relatively fewest gait deviations. Trunk and pelvis kinematics in all planes varied among the participants, but the range of motion during the entire gait cycle in each subject was relatively small. Six children showed less hip extension in late stance, of which 3 had hip flexion contractures of 10° and 3 did not utilize their full hip extension (Fig. 5). One of the children, subject 26, who showed less hip extension during stance due to hip flexion contracture of 10°, had anterior pelvic tilt of nearly 40°. During swing, her knee flexion was limited by her available flexion range of motion of 20°. At initial contact, her knee joint was slightly hyperextended and the knee flexion/extension moment was altered with absence of the first peak knee extension moment (Figs. 5 and 6, pink dashed line). Variation was found in the sagittal plane moments among the subjects, particularly in the knee joint (Fig. 6).

### Comparison of groups

The GDI was lower in all AMC groups compared to the control group, with the lowest in AMC1. Both AMC2 and AMC1 showed greater trunk lateral sway and rotation than AMC3 and the control group. AMC1 had approximately two times greater pelvic rotation than the other groups. All AMC groups showed greater anterior pelvic tilt and less hip extension than the control group. AMC3 showed greater knee flexion during midstance and had greater dorsiflexion during stance than the control group. All AMC groups had less plantarflexion than the control group (Table 2).

**Table 2:** Gait Deviation Index, kinematics, joint kinetics, and time and distance parameters in groups

	AMC 1	AMC 2	AMC 3	Control group	*P*-value
	(*n* = 5)	(*n* = 10)	(*n* = 11)	(*n* = 37)	Kruskal-Wallis
Gait Deviation Index	51.0 [48.4, 59.5] A2, A3, C	76.7 [62.0, 83.1] A1, C	77.7 [70.1, 86.5] A1, C	98.5 [95.1, 106.0] A1, A2, A3	<0.001
Kinematics (°)					
Trunk					
Tilt ant (average) ^a)^	1.6 [-1.9, 2.4]	0.4 [-1.7, 3.5]	−1.4 [-5.7, 1.3]	−0.8 [-4.9, 1.9]	0.372
Lateral sway (range)	16.4 [13.4, 26.0] A3, C	10.6 [6.6, 16.9] A3, C	4.8 [3.7, 7.5] A1, A2	4.3 [3.3, 5.3] A1, A2	<0.001
Rotation (range)	20.6 [17.7, 27.9] A3, C	13.9 [8.5, 22.5] A3, C	6.3 [5.2, 10.8] A1, A2	6.8 [5.2, 8.2] A1, A2	<0.001
Pelvis					
Tilt ant (average)	25.0 [21.8, 28.9] A2, C	15.8 [11.9, 21.8] A1, C	18.5 [14.0, 23.3] C	10.0 [7.0, 14.3] A1, A2, A3	<0.001
Elevation (range)	14.2 [9.5, 22.5] C	11.5 [7.9, 17.1] A3	7.6 [6.6, 9.0] A2	10.0 [7.0, 11.8] A1	0.030
Rotation (range)	31.9 [24.1, 39.3] A2, A3, C	17.4 [10.0, 23.4] A1	15.5 [13.9, 18.6] A1	15.1 [8.8, 19.6] A1	0.008
Hip					
Extension (max)	−3.7 [-20.9, 0.4] C	2.0 [-4.5, 14.7] C	−7.6 [-9.5, 7.1] C	12.5 [6.9, 16.7] A1, A2, A3	<0.001
Flexion (max in swing)	55.0 [49.5, 59.2] A2, A3, C	43.5 [36.5, 49.6] A1, C	47.1 [36.9, 49.8] A1, C	33.5 [29.9, 38.7] A1, A2, A3	<0.001
Abduction (max)	17.8 [16.3, 26.2] A2, A3, C	7.5 [5.8, 12.6] A1	8.0 [5.6, 13.5] A1	7.8 [5.4, 9.4] A1	0.004
Knee					
Flexion (initial contact) ^b)^	17.3 [9.1, 23.9] A3, C	2.8 [-4.3, 15.1]	3.8 [-1.7, 12.1] A1	4.1 [2.2, 6.9] A1	0.038
Flexion (min in midstance) ^b)^	15.9 [6.3, 24.0] C	−2.0 [-8.2, 14.1]	6.8 [1.1, 17.6] C	1.0 [-3.6, 5.6] A1, A3	0.006
Flexion (max in swing)	17.9 [10.7, 25.3] A2, A3, C	59.8 [41.2, 64.7] A1	61.4 [58.6, 62.1] A1	58.3 [54.7, 62.6] A1	0.004
Ankle					
Dorsiflexion (max)	11.3 [9.7, 12.8] A3	13.9 [8.1, 19.1]	19.0 [15.7, 23.0] A1, C	13.2 [9.2, 15.9] A3	0.002
Plantarflexion (max) ^c)^	−4.6 [-6.7, -1.6] A2, A3, C	2.0 [-0.7, 7.3] A1, C	4.3 [-0.1, 11.1] A1, C	16.6 [12.7, 20.2] A1, A2, A3	<0.001
External foot progression (average) ^d)^	25.2 [-3.1, 31.1]	0.7 [- 4.8, 8.6]	0.8 [-3.7, 9.3]	2.7 [0.0, 7.5]	0.163
Joint Moments (Nm/kg)					
Hip					
Flexion (max)	0.367 [0.236, 0.579] A2, A3, C	0.899 [0.684, 1.204] A1	0.954 [0.702, 1.009] A1	0.930 [0.780, 1.160] A1	0.006
Abduction (average stance)	0.176 [-0.054, 0.282]	0.224 [0.056, 0.372]	0.257 [0.203, 0.325]	0.301 [0.264, 0.393]	0.058
Knee					
Extension (max)	0.550 [0.351, 0.682]	0.529 [0.395, 0.795]	0.543 [0.471, 0.816]	0.565 [0.453, 0.713]	0.955
Flexion (max in mid-stance)	0.222 [0.064, 0.532]	0.235 [0.061, 0.412]	0.064 [-0.023, 0.226] C	0.300 [0.175, 0.408] A3	0.035
Valgus (average stance)	0.247 [-0.043, 0.262]	0.071 [0.020, 0.205]	0.143 [0.106, 0.184]	0.163 [0.108, 0.213]	0.196
Ankle					
Dorsiflexion (max)	0.437 [0.129, 0.494]	0.298 [0.218, 0.447] C	0.259 [0.180, 0.328]	0.200 [0.180, 0.252] A2	0.031
Plantarflexion (max)	1.127 [0.794, 1.250] C	1.112 [0.782, 1.275] C	1.260 [1.033, 1.558]	1.320 [1.195, 1.440] A1, A2	0.015
Work (J/kg)					
Hip					
Positive work	0.185 [0.127, 0.375]	0.284 [0.183, 0.397] C	0.218 [0.181, 0.348] C	0.170 [0.110, 0.210] A2, A3	0.008
Negative work	0.082 [0.041, 0.162]	0.171 [0.098, 0.191]	0.148 [0.049, 0.176]	0.130 [0.073, 0.180]	0.403
Knee					
Positive work	0.022 [0.013, 0.049] A2, A3, C	0.103 [0.049, 0.203] A1	0.089 [0.058, 0.157] A1	0.100 [0.060, 0.118] A1	0.014
Negative work	0.035 [0.021, 0.074] A2, A3, C	0.283 [0.214, 0.344] A1	0.276 [0.199, 0.339] A1	0.240 [0.150, 0.300] A1	0.001
Ankle					
Positive work	0.044 [0.023, 0.058] A2, A3, C	0.114 [0.085, 0.138] A1, A3, C	0.247 [0.199, 0.320] A1, A2	0.245 [0.163, 0.290] A1, A2	<0.001
Negative work	0.058 [0.024, 0.081] A2, A3	0.108 [0.075, 0.141] A1, A3	0.178 [0.117, 0.198] A1, A2, C	0.080 [0.053, 0.110] A3	<0.001
Time and distance					
N step length	0.72 [0.64, 0.91]	0.86 [0.77, 0.92]	0.80 [0.71, 0.85]	0.81 [0.75, 0.86]	0.186
N stride length	1.43 [1.31, 1.75]	1.71 [1.58, 1.92]	1.61 [1.47, 1.72]	1.64 [1.51, 1.72]	0.089
N cadence	0.41 [0.35, 0.49] A2, A3, C	0.56 [0.54, 0.59] A1	0.57 [0.55, 0.60] A1	0.59 [0.56, 0.64] A1	0.004
N walking speed	0.291 [0.239, 0.434]	0.498 [0.446, 0.523]	0.465 [0.408, 0.490]	0.500 [0.445, 0.525]	0.060

Median [interquartile range] of Gait Deviation Index, trunk and pelvis kinematics, and lower limb joint angles, moments and work, and non-dimensionalized step length, stride length, cadence, and walking speed in AMC1, AMC2, AMC3 and in the control group. The text following the interquartile range indicates statistically significant difference from A1: AMC1, A2: AMC2, A3: AMC3 and C: the Control group according to post-hoc analysis with a Mann–Whitney *U* test

^a)^ - = posterior, ^b)^ - = extension, ^c)^ - = dorsiflexion, ^d)^ - = internal

Maximum hip flexion moment was lower in AMC1 than in the other groups. Maximum knee extension moment did not differ between the groups but maximum knee flexion moment in midstance was lowest in AMC3. AMC2 showed a lower average knee valgus moment than the other groups, though not significantly. Plantarflexion moment was lower in AMC1 and AMC2 than in AMC3 and the control group (Table 2).

AMC3 and the control group did most of their positive work at the ankle and the most of their negative work at the knee. AMC1 did the greatest positive and greatest negative work in the hip joint. AMC2 did the most of their positive work at the hip joint and most of their negative work at the knee joint (Table 2).

**Fig. 7:**
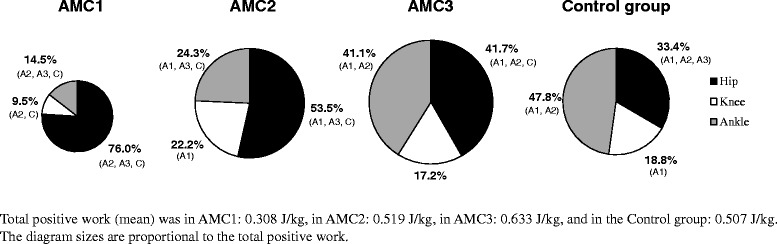
Joint work in children with arthrogryposis. Contributions from the hip, knee and ankle joint to total positive work (mean percent) in AMC1, AMC2 and AMC3, and Control groups. Text in parentheses indicates statistically significant difference from A1: AMC1, A2: AMC2, A3: AMC3, and C: the Control group

The hip’s contribution to total positive work differed significantly between the groups, wherein all AMC groups had greater than the control group, with the greatest in AMC1 followed by AMC2 and then AMC3. The knee’s contribution to total positive work differed significantly between the groups, with the lowest in AMC1. The ankle’s contribution to total positive work differed significantly between the groups, wherein AMC1 had lower than any other groups (Fig. 7).

The cadence was lower in AMC1 than in all other groups. The walking velocity was lower in AMC1 than in AMC2 and the control group. The step length and stride length did not differ significantly between the groups (Table 2).

### Performed orthopedic surgery

Orthopedic surgical corrections of deformities in the lower limbs had been performed on 23 children. Two children had undergone spine surgery for correction of scoliosis. There was no significant difference between groups in number of performed orthopedic procedures (*p* = 0.185). Performed surgery in lower limb and spine for each subject are shown in Table 3.

**Table 3:** Age (years) at the time of study participation, orthopaedic procedures, and side and age at performed surgery

Subject	Age	Orthopaedic procedures and age at performed surgery
1	5.2	Ponseti + Achilles tenotomy bi [0.5y]; Ponseti + Achilles tenotomy and dorsal capsulotomy bi [1y]; Ponseti + Achilles tenotomy bi [3y]
2	5.4	Quadriceps lenghtening bi, vertical talus R [3y]; Open hip reduction with femoral derotation and varus osteotomy, pelvic osteotomy R [4y]
3	16.6	Club foot bi [1y]; Pelvic osteotomy R [6y]; Pelvic osteotomy L [9y]
4	16.9	Club foot bi [1y]; Correction osteotomy foot bi, Extension osteotomy distal femur bi [6y]; Guided growth (plate and screws) distal femur bi [11y]
5	17.8	Club foot bi [<1y]; Hamstring lengthening bi [5y]; Lengthening of flexor hallucis longus and capsulotomy R [15y]
6	5.0	Ponseti L, Redression of vertical talus and knee dislocation R, Achilles tenotomy bi [<0.5y]; Club foot L, Vertical talus R, Extension osteotomy distal femur L, Quadriceps lengthening R [1y]; Achilles lengthening, dorsal capsulotomy bi [4y]
7	5.7	Ponseti [<0.5y]; Achilles tenotomy, tibialis anterior transfer bi [3y]
8	5.7	Open reduction hip L, vertical talus R [<1y]; Vertical talus L [1.5y]
9	6.0	Vertical talus bi [3.5y]
10	6.1	Ponseti + Achilles tenotomy bi [<1y]; Club foot bi, Open reduction hip R [1y]; Club foot, decancellation of cuboid and tibialis anterior transfer bi [2.5y]
11	8.8	Vertical talus bi [5y]
12	10.4	Ponseti + Achilles tenotomy R [<0.5y]; Excision of pterygium + lengthening skin plasty, Achilles lengthening, dorsal capsulotomy, tibialis anterior transfer R [7.5y]
13	13.2	Insertion of VEPTR for correction of scoliosis [7y]; Three expanions of VEPTR [8 - 9.5y]; Achilles lengthening, dorsal capsulotomy R, Spine fusion [10.5y]
14	13.8	Talectomy L [1y]; Talectomy R [1.5y]
15	16.7	Achilles tenotomy bi, Toe flexor tenotomy L [<0.5y]; Club foot bi [1y]; Club foot and cuboid osteotomy L [3y]
16	5.3	Achilles tenotomy, oblique talus [1.5y]
17	6.5	Quadriceps lenghtening bi, Achilles tenotomy R [1y]
18	8.2	Excision of pterygium + skin lengthening plasty, Achilles lengthening bi [<1y]; Achilles lengthening, skin lengthening plasty bi [3y]
19	8.7	Club foot bi [1y]
20	10.1	-
21	11.9	Open reduction hip bi, Club foot L [1.5y]
22	12.2	-
23	13.2	Open reduction hip and femoral varus osteotomy L [2.5y]; Open reduction hip and femoral varus osteotomy R [3y]; Femoral valgus osteotomy [6.5y]
24	14.6	Spine fusion [13.5y]
25	15.1	Guided growth (plate and screws) distal femur bi, Achilles lengthening, dorsal capsulotomy R [12.5y]
26	16.6	Pelvis osteotomy R, femoral osteotomy L [1y]; Pelvis and femoral osteotomy L [9.5y]; Pelvis and femoral osteotomy R [10.5y]; Femoral physiodesis R [11.5y]

*Bi* bilaterally, *y* years, *R* right, *L* left, *VEPTR* vertical expandable prosthetic titanium rib

## Discussion

AMC is a group of heterogeneous disorders characterized by multiple joint contractures [18]; in our study, we have illustrated the diversity of gait in children with AMC, but have not been able to identify overall characteristic gait patterns in this patient population. Variations due to their individual requirements are common. We have instead tried to classify the participants into subgroups depending on need for joint stabilization, and to describe gait dynamics in their highest possible level of ambulation. Wheelchair use was reported in 12/26 children as a complement to walking, including the entire AMC1 group, which relates to previous findings of higher energy effort during walking in children with AMC [9]. Hip flexion contracture of less than 20°-30° and active hip motion have been reported as important factors for independent or community ambulation [5, 7]. None of the children in our study group had hip flexion contractures greater than 20°, and all children had hip extensor strength of grade 4–5 except two in AMC1. In a group of children walking without orthoses, excessive trunk lean and pelvic elevation have been proposed as compensation for limited hip flexion movements and strength [19]. In our study group hip flexion weakness was found in 6/10 limbs in AMC1. Correlation between excessive ipsilateral trunk sway and reduced internal hip abduction moment has been reported in children with AMC walking barefoot [12] and was attributed as a compensatory solution for weak hip abductors [12], as previously been reported in children with myelomeningocele [20]. To improve quality of gait function and achieve a more efficient gait, the importance of hip joint stability has been advocated [4]. None of the children in our study group had dislocated hips at the time of gait analysis, and hip surgery had been performed in seven children.

In our study group, the foot and ankle joints were most frequently involved, which is in accordance with Bevan et al. [3]. Different types of orthotic ankle joints were used to compensate for plantarflexor weakness and stabilize the ankle, but also according to each individual’s acceptance, resulting in the observed spectrum of dorsi/plantarflexion moments. Use of a carbon fiber spring ankle joint has been shown to increase plantarflexion moment in children with plantarflexor weakness [10]. Six children, distributed into AMC1 and AMC2 used orthoses with carbon fiber spring ankle joints.

A dominating attribute in AMC1 is knee extensor weakness; KAFOs with locked knee joints were therefore used to stabilize the knees. We attribute this group’s excessive frontal and transverse trunk and pelvic movements as compensatory solutions for locked orthotic knee joints to advance the leg and clear the foot during swing. This gait pattern reduces the hip abduction moments. Hip flexion moment was low, which corresponds to their lack of hip extension during late stance in combination with an exaggerated pelvic tilt. Two children in this group had hip flexion contractures and three children did not utilize their full hip extension, which might be a gait strategy when walking with bilateral locked KAFOs. Use of walkers to maintain balance has been described in children walking with locked KAFOs bilaterally [21]. All such children in the present study, however, preferred to walk without a walking aid. This also highlights the importance that orthoses/shoes should be tailored to compensate for each individual’s contracture. During stance, the children’s knees were restricted in a position corresponding to their knee flexion contractures of 10°–30°. Their knee extension/flexion moments are therefore entirely due to orthoses.

The main attribute in AMC2 is plantarflexor weakness and therefore KAFOs with open knee joints or AFOs were used to stabilize the ankles. Knee varus instead of valgus moment was seen in two children using AFOs, of which one had hip abductor weakness and thereby also displayed stance hip adduction moment. The treatment concept of KAFOs with open knee joints is to stabilize the knee in frontal and transversal planes while not restricting knee flexion. Both children had used KAFO-O previously but in recent years preferred AFOs. Hip flexion moment was similar to the control group but three children with less hip extension in late stance in combination with exaggerated pelvic tilt showed lower hip flexion moment. The similar knee extension/flexion moment to the control group is likely due to orthoses stabilizing the ankle joint in the sagittal plane and knee flexion contractures less than 15°.

In AMC3, hip flexion moment was similar to that of the control group despite less hip extension during late stance and greater pelvic tilt. AMC3 had considerably lower knee flexion moment during midstance than other groups, corresponding to their greater passive dorsiflexion and corresponding lack of full knee extension. They had similar ankle moments as the control group, as they walked with only shoes and included only one child with some plantarflexor weakness.

In normal gait propulsion, approximately half of the positive work in the lower limbs is provided at the ankles [20, 22]. In the present study, all AMC groups displayed a proximal work shift, wherein the hip contributed more than the ankle to overall positive work. This was more pronounced in those walking with orthoses and particularly locked orthotic knee joints. The work analysis demonstrates that the children rely heavily on their hip muscles, and based on their trunk movements, presumably their core muscles to provide propulsion. AMC3 furthermore, had somewhat higher total positive work than TD children, which can be attributed to higher hip moments and higher total negative work in AMC3 but a similar walking speed to TD children.

The heterogeneity among the participants regarding joint contractures, muscle weakness and different types of orthoses used in this study might seem a limitation, but as it reflects the complexity of the condition, it was unavoidable. It could be also considered a study limitation that children with AMC were tested in orthoses or shoes rather than barefoot, but this choice was made deliberately; we have collected gait data on those who could walk barefoot, but the ambulation level was often lower or nonexistent. Children in AMC1 had no walking ability without orthoses and five of the children in AMC2 had a lower ambulation level without orthoses. We therefore analyzed what we believe is their optimal and typical gait. It may have been optimal if children in the control group had also walked with shoes, as some gait parameters change slightly with shoes [23]. In the present study, however, the magnitude of gait parameters differences between AMC and control group were quite large, and we therefore believe there is little risk of over-interpreting small differences accounting to shoe use. It should also be noted that while we computed the GDI, we only used it to select which side to analyze. Since GDI does not incorporate upper body movements, it probably underestimates the true deviation in this population.

## Conclusion

Children with AMC have potential to achieve functional ambulation despite a wide spectrum of muscle weakness and joint contractures, with orthotic solutions ranging from locked KAFOs to AFOs to shoes only. While all children with AMC had gait deviations, the highest deviations were observed in children requiring the most extensive orthotic support. Their kinetic patterns indicate how children with a high degree of joint deformity and muscle weakness are able to walk. The hip contributed more to positive joint work in all AMC groups, particularly in AMC1, than in TD children. For optimal use of muscle strength, it is important to minimize hip flexion contractures, and preserve hip muscle strength in order to attain sufficient forward propulsion during walking. We believe that each child, based on his/her joint contractures, muscle weakness, and need for external stabilization, has developed an optimal and efficient gait.
